# Combined use of intranasal Dexmedetomidine and an oral novel formulation of Midazolam for sedation of young children during brain MRI examination: a prospective, single-center, randomized controlled trial

**DOI:** 10.1186/s12871-022-01897-x

**Published:** 2022-11-23

**Authors:** Hongbin Gu, Liyan Miao, Jie Bai, Guolin Lu, Qian Lei, Lijun Yang, Denggui Wang

**Affiliations:** 1 Department of Anesthesiology, Shanghai Children’s Medical Center, School of Medicine, Shanghai Jiao Tong University, Shanghai, China; 2 Department of Anesthesiology, Fujian Children’s Hospital (Fujian Branch of Shanghai Children’s Medical Center), College of Clinical Medicine for Obstetrics & Gynecology and Pediatrics, Fujian Medical University, Fuzhou, China; 3 Department of Anesthesiology, Fujian Maternity and Child Health Hospital, College of Clinical Medicine for Obstetrics & Gynecology and Pediatrics, Fujian Medical University, Fuzhou, China

**Keywords:** Oral, Dexmedetomidine, Midazolam, Pediatrics, Brain MRI, Sedation

## Abstract

**Background:**

To evaluate the safety and effectiveness of different dosages of intranasal Dexmedetomidine (DEX) in combination with oral midazolam for sedation of young children during brain MRI examination.

**Methods:**

Included in this prospective single-blind randomized controlled trial were 156 children aged from 3 months to 6 years and weighing from 4 to 20 Kg with ASA I-II who underwent brain MRI examination between March 2021 and February 2022. Using the random number table method, they were divided into group A (using 3 ug/kg intranasal DEX plus 0.2 mg/Kg oral midazolam) and group B (using 2 ug/kg intranasal DEX plus 0.2 mg/Kg oral Midazolam). The one-time success rate of sedation, sedation onset time, recovery time, overall sedation time, and occurrence of adverse reactions during MRI examination were compared between the two groups. The heart rate (HR), mean arterial pressure (MAP), and percutaneous SpO_2_before and after drug administration were observed in both groups. Differences in sedation scores between the two groups were compared before intranasal drug administration (T0), 10 min after drug administration (T1), at the time of falling asleep (T2), at the end of examination (T3), and at the time of recovery (T4).

**Results:**

The one-time success rate of sedation in group A and B was 88.31% and 79.75% respectively, showing no significant difference between the two groups (*P*>0.05). The sedation onset time in group A was 24.97±16.94 min versus 27.92±15.83 min in group B, and the recovery time was 61.88±22.18 min versus 61.16±28.16 min, both showing no significance difference between the two groups (*P*>0.05). Children in both groups exhibited good drug tolerance without presenting nausea and vomiting, hypoxia, or bradycardia and hypotension that needed clinical interventions. There was no significant difference in the occurrence of abnormal HR, MAP or other adverse reactions between the two groups (*P*>0.05).

**Conclusion:**

3 ug/kg or 2 ug/kg intranasal DEX in combination with 0.2 mg/kg oral Midazolam both are safe and effective for sedation of children undergoing MRI examination with the advantages of fast-acting and easy application.

**Trial registration:**

It was registered at the Chinese Clinical Trial Registry (ChiCTR1800015038) on 02/03/2018.

## Background

Magnetic resonance imaging (MRI) is frequently used for the examination of children with brain diseases. It is difficult or even impossible to perform the examination in most children less than 6 years of age. To overcome this difficulty, it is often necessary to perform clinical sedation or general anesthesia to ensure a successful examination of MRI. Due to the cost of general anesthesia, MRI examination assisted by sedation is a simple and effective method and has become a new direction of research in this field [1, 2].

The general principle of drug administration for sedation-assisted examination in young children should be harmless, non-invasive, and simple to be accepted by both children and their parents or guardians. Oral chloral hydrate in combination with intranasal Dexmedetomidine (DEX) is a common clinical practice [3]. However, chloral hydrate is strongly irritable and likely to increase gastrointestinal reactions and other adverse reactions. In addition, the cardiac and neurological toxicities associated with chloral hydrate have also aroused increased attention and concern in recent years, and therefore it is not recommended for use in clinical practice, especially in young children. Oral Midazolam is a commercial drug recently available in mainland China and is acceptable by children because of its sweet taste, high effectiveness and safety, thus avoiding emotional stress and anxiety due to fear of intravenous injections or long-term psychosomatic influence on the children, and at the same time reducing agonies of the parents. MRI examination needs to have a relatively long duration of sedation and avoid “being arousal” during the process of examination. The half-life time of Midazolam is about 2 hours, which is appropriate for completing the MRI examination. We hypothesized that the pharmacodynamics of medicine combined with an oral novel formulation was not similar to the traditional intravenous formulation [4, 5]. Based on the above understanding about Midazolam, we designed this study, aiming to evaluate the effectiveness and safety of using the recommended minimum dose of oral Midazolam in combination with intranasal DEX for sedation during MRI examination in young children, knowing that obtainment of a definite conclusion has great clinical reference and popularization significance.

## Materials and Methods

### Patient selection

This prospective single-center single-blind randomized controlled trial was performed by the Declaration of Helsinki and good clinical practice guidelines, approved by the ethics committee of our hospital (SCMCIRB-K20170690), and registered at the Chinese Clinical Trial Registry (ChiCTR1800015038) before the subject enrollment. Included in this study were 156 children aged from 3 months to 6 years and weighing from 4 to 20Kg with ASA I-II who underwent brain MRI examination in Fujian Hospital of Shanghai Children’s Medical Center between May 2021 and February 2022. Using the random number table method, they were divided into group A (using 3 ug/kg intranasal DEX plus 0.2 mg/Kg oral Midazolam) and group B (using 2 ug/kg intranasal DEX plus 0.2 mg/Kg oral Midazolam).

**Fig 1:**
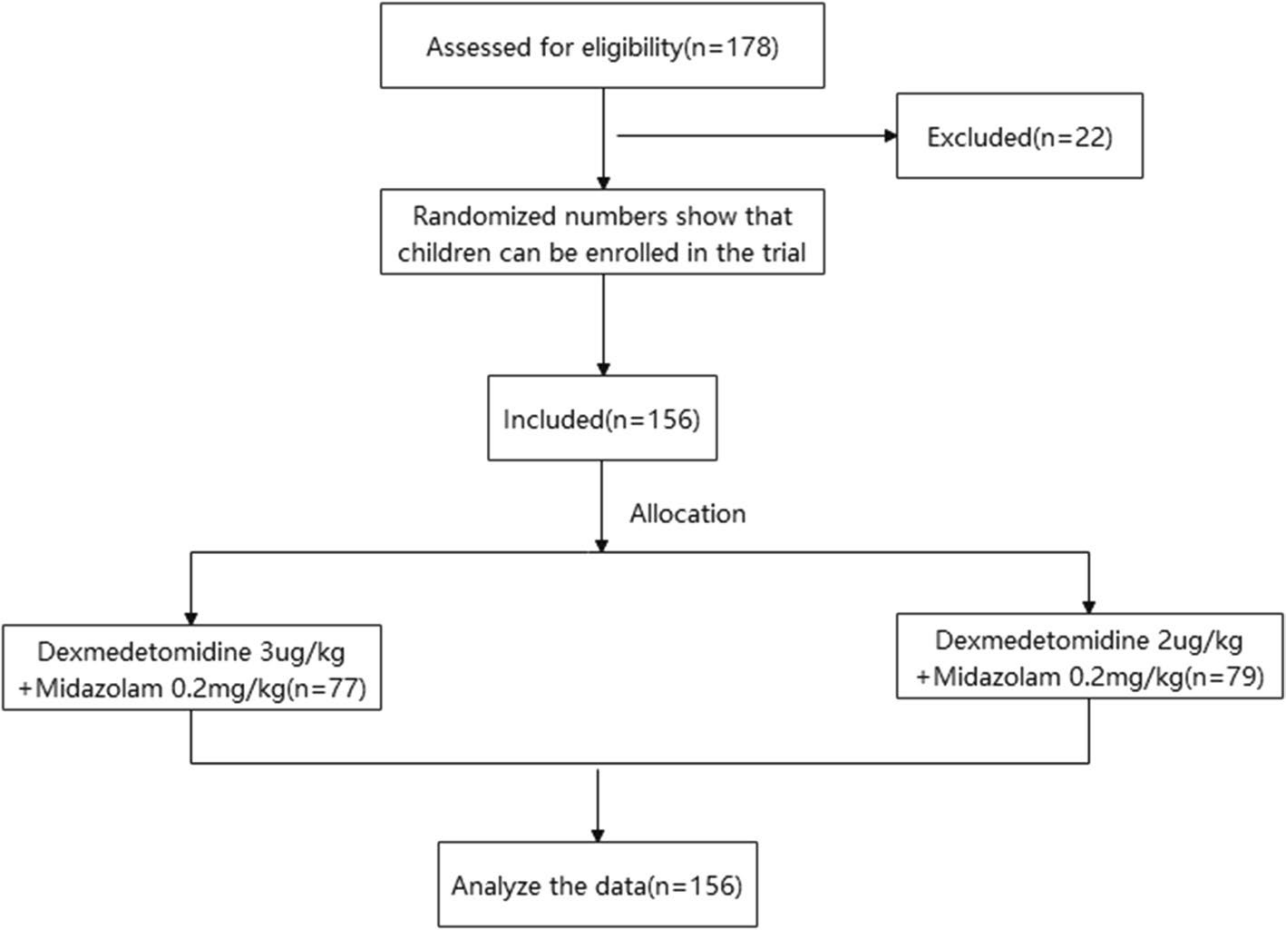
Flow chart of the experiment

Inclusion criteria: examination time within 60min; no upper respiratory tract infection in the recent two weeks; and the parents or guardians of the included children agreeing to sign informed consent. Exclusion criteria: allergic history of related drugs; patients with primary coronary heart disease (CHD) and acute gastroenteritis; patients with a history of receiving sedative and hypnotic drugs within the previous 48 hours; patients with a body mass index (BMI) ≥28); patients unable to take oral drugs; patients with respiratory tract infection or nasal catarrhal symptoms; and patients with a history of paradoxical reactions for Midazolam, existing bradycardia and/or hypotension. The experimental procedure is shown in Fig. 1.

### Methods

#### Sedation method

Before sedation, the parents or guardians of the included children were advised to routinely fast their babies from fluid for 2 hours, avoid breastfeeding for 4 hours and fast their babies from solid food for 6 h, without strictly implementing the strategy of sleep deprivation. On the very day of sedation, the child was assessed by the anesthesiologist, including the demographic data, current history, diagnosis, allergy, and ASA classification. Before sedation, the children were randomized into group A and B by using the random number table method. After obtaining informed consent from the parents or guardians, the drugs were administered by the appointed nurse of the sedation room, who was also responsible for recording the observational parameters. Children in group A were first administered with 0.2 mg/kg oral Midazolam solution (2 mg/ml, Batch No. 0L912011, Yichang Renfu Pharmaceuticals, Yichang, China) followed by 3ug/kg intranasal DEX injection solution in two equally divided doses through both nostrils (0.1mg/ml, Batch No. 21033131, Yangzijiang Pharmaceuticals Group, Taizhou, China); after drug administration, the nasal cavities were gently massaged externally with the child laid flat for 1-2 min. Drug administration in group B was carried out in the same way except that the dose of DEX was 2 ug/Kg. After drug administration, drug acceptance by the children, observational parameters, and sedation scores were recorded. At the same time, sedation onset time, recovery time, overall sedation time, and possible adverse reactions were all recorded. The degree of sedation was assessed by Ramsay Scale (Table 1) at 10-min intervals. When the Ramsay Scale score was ≥ 5, a brain MRI examination could be started. To reduce the noise from the MRI machine, ear plugs were used for the children during the examination. Completion of the examination by one entry into the machine was defined as success of sedation. Sedation onset time exceeding 30min, Ramsay score ≤ 5, or the child waking up during the process of examination was defined as failure of sedation. If such a case occurred, the family would be advised to do the sedative examination on a selective day, or select 50 mg/kg chloral hydrate by enema for supplementary sedation. After the examination, the child would be sent to the recovery room for observation of consciousness, SpO2, and HR; when the Modified Aldrete Score (MAS) was ≥9 (Table 2), the child was allowed to leave the hospital.

**Table 1:** Ramsay sedation score

Score	Response
1	Awake, anxious, agitated, restless
2	Awake, cooperative, tranquil
3	Responds to commands
4	Asleep, brisk response to stimulus
5	Asleep, sluggish response to stimulus
6	Asleep, no response to stimulus

**Table 2:** Modified Alderete score

Score	Response
**Breathing**	
2	Able to breathe deeply
1	Dyspnea
0	Apnea
**Circulation**	
2	Systemic blood pressure<20% of the preanesthetic level
1	Systemic blood pressure between 20%–49% of the preanesthetic level
0	Systemic blood pressure ≥ 50% of the preanesthetic level
**SpO2**	
2	Maintaining O2 saturation>92% on room air
1	Needing inhalation to maintain O2 saturation>92%
0	O2 saturation<92% despite O2 supplementation
**Consciousness**	
2	Fully awake
1	Arousable
0	Not responding
**Mobility**	
2	Able to move four extremities on command
1	Able to move two extremities on command
0	Able to move zero extremities on command

The one-time successful rate of sedation was considered as the primary outcome. One-time successful sedation is defined as Ramsay ≥ 5 points and completion of the examination by one entry into the machine after the initial administration. Sedation scores at the different time points of observation, onset time, recovery time, and overall sedation time were regarded as secondary outcomes.

### Monitoring

The following data and information were recorded: (1) demographic data of the patients in both groups, including sex, age, and weight; (2) vital signs before and after drug administration, including HR, MAP, and SpO_2_; (3) the adverse reaction rate, one-time success rate of sedation, sedation onset time (from drug administration to reaching Ramsay ≥5), recovery time (from satisfactory sedation to reaching MAS ≥9), and overall sedation time (from drug administration to reaching MAS ≥9); (4) sedation onset time exceeding 30min, waking up during examination, or Ramsay score ≤5 was defined as sedation failure; (5) occurrence of adverse reactions: HR lower or higher than 20% of that before sedation was defined as abnormal HR; blood pressure (BP) higher or lower than 20 of that before sedation was defined as abnormal BP; SpO2<90% was defined as hypoxia; nausea and vomiting.

### Statistical methods

We estimated the sample size according to the success rate of sedation in group A and group B . By consulting the literature and previous pre-experimental results, we set the success rate of group A as 90%, the success rate of group B as 75%, α =5%, 1-β =0.80, and the degree of freedom to1. The estimated sample size was 97, and as about 30% of samples may be lost, the study was expected to include at least 127 patients. Finally, 156 patients were included for analysis in this study SPSS 26 was used to perform statistical analysis. Measurement data were verified for normality by the Kolmogorov-Smirnov test. Measurement data of normal distribution are expressed as the mean ± standard deviation (SD). Comparison between the two groups was verified by t-test. Measurement data of abnormal distribution are expressed as median (interquartile range). Comparison between two groups was verified by Kruskal-Wallis (K-W) test. Changes in the sedation score over time were analyzed using a mixed-effect model with repeated measurement (MMRM) analysis using sedation scores at all follow-up time points as the dependent variable, treatment as the main factor, sedation scores at T0 as a covariate, and random intercept to model within-subject correlation. Enumeration data are expressed as a percentage (%) and were verified by Pearson x^2^ or Fisher exact probability test. P<0.05 was considered statistically significant.

## Results

### Demographic data of the patients

GroupA included 77 cases and group B included 79 cases, totaling 156 cases. The diagnoses of patients in group A (group B) included brain developmental delay 22 (19), intracranial tumors 8 (11), cerebrovascular malformations 1 (0), intracranial infections 6 (8), epilepsy 7(8), traumatic brain injury 4 (7), and others 29 (26), respectively. The demographic data (sex, BMI, and weight) of the 156 pediatric patients are listed in Table 3, the duration of the examination, and the diagnoses of patients showing no significant difference between the two groups (>0.05).

**Table 3:** Demographic data of the patients(*N*=156)

Group	*n=*	Male/Female	Age (month,‾x±SD)	Weight (kg,‾x ±SD)	The duration of examination (minute, x ±SD)
A-Group	77	48/29	30.34±15.67	13.04±3.14	35.50±8.65
B-Group	79	56/23	27.68±17.51	12.72±3.68	38.50±12.45
χ^2^		1.282			
*t*			0.997	0.585	0.645
*p*		0.257	0.109	0.111	0.350

Notes: All the data were normal distribution, and expressed as the mean ± standard deviation (SD). Enumeration data are expressed as a number.

### Sedation outcomes

The one-time sedation success rate after drug administration was observed as follows: 88.31% in group A and 79.75% in group B. Although the one-time sedation success rate in group A was 8.6% higher than that in group B, statistical analysis showed no significant difference between them (P>0.05) (Table 4).

**Table 4:** Comparison of the one-time sedation success rate between group A and group B

Group	*n=*	Success (*n*=)	Failure (*n*=)	Success rate (%)
A-Group	77	68	9	88.31
B-Group	79	63	16	79.75
χ^2^				2.126
*p*				0.145

Note: All enumeration data were expressed as numbers or percentages. Groups A: 3 ug/kg intranasal DEX plus 0.2 mg/kg oral Midazolam solution (2 mg/ml); Group B: 2 ug/kg intranasal DEX plus 0.2mg/kg oral Midazolam solution (2 mg/ml).

### Ramsay score

Ramsay scores at T0, T1, T2, T3 and T4 were compared between the two groups, showing no significant difference between the two groups (*P*>0.05) (Table 5).

**Table 5:** Ramsay scores at different time points

Group	*n*=	T0(‾x±SD )	T1 (‾x±SD )	(‾x±SD )	T3(‾x±SD )	T4(‾x±SD )
A	77	1.05±0.22	3.74±1.22	5.05±0.22	3.88±1.11	2.19±0.40
B	79	1.06±0.25	3.56±1.38	5.04±0.19	3.57±1.20	2.15±0.36
*p*	0.25					

Notes: T0 (before intranasal drug administration), T1 (10 min after drug administration), T2 (at the time of falling asleep), T3 (at the end of examination), T4 (at the time of recovery). All the data were normal distribution and expressed as the mean ± standard deviation (SD). As can be seen from the table, there is no statistical difference between the Ramsay scores of the two groups at all time points.

### Sedation onset time

There was no significant difference in sedation onset time, recovery time and overall sedation time between the two groups (*P*>0.05) (Table 6).

**Table 6:** Comparison of sedation onset time, recovery time and overall sedation time between group A and B

Group	***n=***	Sedation onset time (‾x±SD, min)	Recovery time (‾x±SD, min)	Overall sedation time (‾x±SD, min)
A	77	24.97±16.94	61.88±22.18	86.86±27.26
B	79	27.92±15.83	61.16±28.16	89.09±32.00
*t*		-1.124	0.177	-0.468
*p*		0.361	0.692	0.533

Note: All the data were normal distribution, and expressed as the mean ± standard deviation (SD). All enumeration data were expressed as a number. Groups A: 3 ug/kg intranasal DEX plus 0.2 mg/kg oral Midazolam solution (2 mg/ml); Group B: 2 ug/kg intranasal DEX plus 0.2 mg/kg oral Midazolam solution (2 mg/ml)

### Adverse events

No hypoxia, nausea, or vomiting was observed in either group during the peri-sedation period. HR after sedation was 20% lower than that before sedation in 12 cases in group A versus 10 cases in group B; MAP after sedation was 20% lower than that before sedation in 8 cases in group A versus 5 cases in group B, showing no significant difference between the two groups (*p*>0.05) (Table 7).

**Table 7:** Comparison of abnormal heart rate occurrence between group A and B

Group	n=	Abnormal HR(n=)	Abnormal MAP (n=)	Occurrence of abnormal HR (%)	Occurrence of abnormal MAP (%)
A	77	12	8	15.58	10.39
B	79	10	5	12.66	6.33
χ^2^				0.276	0.842
*p*				0.6	0.359

*HR* heart rate, *MAP* mean arterial pressure

Note: All enumeration data were expressed as numbers or percentages. groups A: 3 ug/kg intranasal DEX plus 0.2 mg/kg oral Midazolam solution (2 mg/ml); group B: 2 ug/kg intranasal DEX plus 0.2 mg/kg oral Midazolam solution (2 mg/ml).

## Discussion

It was found in our study that oral Midazolam in combination with the use of 3 ug/kg or 2 ug/kg intranasal DEX had a similar sedation effect and showed no significant difference in safety between the two combination regimens. The one-time sedation success rate and sedation onset time were rational and effective in both combinations.

MRI examination usually lasts a relatively long period and produces large noises, which may affect the quality of examination in young children due to crying, anxiety, and fear. To improve the accuracy of imaging diagnosis, it is necessary to use sedatives in preschool children and young children with chronic diseases who need to undergo MRI examination [6].

Midazolam is a type of short-acting benzodiazepine with anti-anxiety, anti-convulsion, sedative, and hypnotic activities [7]. Also, it has a certain respiratory inhibitory effect, depending on the severity of the disease and the dose of the drug. But as it has a minimal effect on the cardiovascular system, it is a commonly used sedative in clinical practice [8], especially in young children. Research finds that Midazolam or a like drug alone is often ineffective [9, 10], and therefore it is often used in combination with other drugs. Oral Midazolam in combination with intranasal DEX can largely increase the success rate of sedation and offers a good sedative outcome. However, many medical institutions prepared the oral Midazolam solution by mixing its intravenous injection form with syrup, but it still tastes bitter and irritable, which often induces nausea and vomiting and therefore is unacceptable by young children. The locally available commercial product of oral Midazolam tastes sweet and is easy to be accepted by young children and complies with the ethical rule.

DEX is an α_2_ adrenoceptor agonist and can induce a state similar to natural sleep [11].

Compared with other sedatives, it has minimal impact on respiration, with a low

occurrence of respiratory depression [12] and a potent sedative effect. Compared with

chloral hydrate, DEX can provide a more effective sedation action [13, 14]. A meta-analysis

showed Intranasal administration of DEX is superior to oral chloral hydrate for sedation

during pediatric CT/MRI examinations and has a better safety profile [15]. Intranasal

administration of DEX has good tolerance and the sedative effect that it produces is

similar to the intravenous injection form [16]. It has therefore been gradually and more

commonly used in clinical practice.

Procedural sedation using the combination of intranasal Dexmedetomidine and ketamine

is associated with acceptable effectiveness, low rates of adverse events, and may shorten

the sedation induction time [17, 18]. However, the instructions of ketamine show that

mental symptoms such as hallucinations, restlessness, and nightmares may occur during

the recovery period of anesthesia. Therefore, further research is required on the mental side effects. Combined use of oral Midazolam and intranasal DEX can offer a high one-time sedation success rate with a good sedative effect, therefore avoiding a second examination, and saving the manpower, financial resources, and time of the family. In addition, the sedated child is easy to recover and the basic parameters remain stable during the process of sedation.

The main result of the present study revealed that the sedation onset time, sedation maintenance time, and moderate-deep sedative effect of combined used of oral Midazolam and intranasal DEX were similar to what was reported in previous studies. Li et al reported their combined use of buccomucosal Midazolam with intranasal DEX during CT examination in children with autism and achieved a 95% examination success rate without the occurrence of respiratory depression or hemodynamic disturbance that needed clinical intervention [19, 20]. Cozzi et al [21] reported an 84% sedation success rate in their 108 children undergoing MRI examination by using 0.5 mg/kg Midazolam plus 3 ug/kg intranasal DEX, Li BL et al. explored the efficacy and safety of using 3 ug/kg intranasal DEX plus 0.2 mg/Kg oral Midazolam [22], believing that Midazolam in combination with intranasal DEX is a safe and effective regimen for sedation. Although they did not observe significant adverse reactions, the dose of the two drugs that they used is significantly larger than that we used in this study, indicating that their regimen is not safe as ours, and therefore should be selected with caution in clinical practice. This discrepancy may be due to the following reasons. First, the age of the children in our study was narrow, while Cozzi ‘s study included children ranging in age from 4 to 209 months. The study participants mentioned by Cozzi were older than ours. Therefore, the required drug dose for DEX is smaller in our research. The second reason should be attributed to the DOR’s formulation. The medication we used this time is an oral formulation rather than the intravenous formulation used in many previous studies, so the absorption effect would be better, and result in a lower dose of medication required. In addition, the present study mainly observed the sedation effect in young children during MRI examination. Although they had various types of diseases, the examiners and technical parameters were relatively fixed, which provided good tacit cooperation between the technicians and therefore indirectly improved the efficiency and success rate of examination. Therefore, our study applied a lower dose of Dexmedetomidine to achieve the same clinical effect. Although the use of two different drugs and drug administrations is more time-consuming and complex as compared with propofol and other narcotics alone, it reduces the risk of respiratory depression and avoids vascular injection. Although the use of a relatively high dose of DEX does not seem to induce significant respiratory depression, it affects hemodynamics [23] and therefore it is necessary to determine an appropriate dose to achieve a satisfactory outcome of sedation. Some studies reported that the dose of intranasal DEX was 1-4 ug/kg. Li et al reported that the ED95 of intranasal DEX used for pulmonary function tests in children aged 1-3 years was 2.64 ug/kg [24]. According to the report by Miller et al [25], 2.5-3 ug/kg intranasal DEX could obtain an even higher success rate of sedation as compared with intranasal inhalation of a low dose (1-2 ug/kg) of atomized DEX for sedation of transthoracic echocardiography (TTE) in pediatric patients with CHD. Given the above findings, we used two regimens (3 ug/kg DEX+0.2 mg/kg Midazolam in group A, and 2 ug/kg DEX+0.2 mg/kg Midazolam in group B) in our study. The results showed no significant difference in sedation onset time and recovery time between the two groups (*P*>0.05). Although the sedation success rate in group A was slightly higher than that in group B, the difference was not statistically significant (P>0.05), probably because the synergistic effect of Midazolam reduced the required dose of DEX.

The effect of the sympathetic nerve block of DEX may reduce HR and BP [26]， but no hemodynamic change that needed clinical intervention occurred in the cohort of patients in our study. In addition, there was no significant difference in the occurrence of abnormal HR between the two groups (*P*>0.05). Weconclude that either 2 ug/kg or 3 ug/kg DEX can be safely used for sedation in young children. No hypoxia, nausea, or vomiting occurred during the sedation period in both groups, indicating that Midazolam and DEX are well tolerated by sick children.

Our study had some limitations. First, there was a lack of specific thresholds for prospective airway and hemodynamic interventions. Second, as we were unable to perform the study in a double-blind manner and did not employ a third party to make the evaluation, subjective deviation could not be avoided completely. Third, this is a single-center study and therefore the findings and conclusions obtained in this study may not be universally significant. Fourth, some retrospective memory of some parents or guardians may produce selective deviation and informative errors which may affect the results of the experiment. Finally, we failed to build up a complete set of drug dose combinations and therefore the result obtained in this study only indicates that the dose combination of the sedative reported herein is safe and effective and does not represent the optimal dose.

In summary, 2 ug/kg or 3 ug/kg DEX +0.2 mg/kg Midazolam can provide a high one-time sedation success rate in young children for brain MRI examination without inducing significant changes in vital signs. A small dose (2 ug/kg) of intranasal DEX and 0.2 mg/kg oral Midazolam should be an even safer compatible dose and worthy of clinical promotion.

## Data Availability

The datasets used and/or analysed during the current study are available from the corresponding author on reasonable request.
